# Outcomes before and after Implementation of the ERAS (Enhanced Recovery after Surgery) Protocol in Open and Laparoscopic Colorectal Surgery: A Comparative Real-World Study from Northern Italy

**DOI:** 10.3390/curroncol31060222

**Published:** 2024-05-21

**Authors:** Lucia Mangone, Federica Mereu, Maurizio Zizzo, Andrea Morini, Magda Zanelli, Francesco Marinelli, Isabella Bisceglia, Maria Barbara Braghiroli, Fortunato Morabito, Antonino Neri, Massimiliano Fabozzi

**Affiliations:** 1 Epidemiology Unit, Azienda USL-IRCCS di Reggio Emilia, 42122 Reggio Emilia, Italy; lucia.mangone@ausl.re.it (L.M.); isabella.bisceglia@ausl.re.it (I.B.); mariabarbara.braghiroli@ausl.re.it (M.B.B.); 2 Surgical Oncology Unit, Azienda USL-IRCCS di Reggio Emilia, 42122 Reggio Emilia, Italy; federica.mereu@ausl.re.it (F.M.); maurizio.zizzo@ausl.re.it (M.Z.); andrea.morini@ausl.re.it (A.M.); magda.zanelli@ausl.re.it (M.Z.); massimiliano.fabozzi@ausl.re.it (M.F.); 3 Biotechnology Research Unit, AO di Cosenza, 87100 Cosenza, Italy; f.morabito53@gmail.com; 4 Scientific Directorate, Azienda USL-IRCCS di Reggio Emilia, 42122 Reggio Emilia, Italy; antonino.neri@ausl.re.it

**Keywords:** enhanced recovery, fast-track surgery, minimally invasive surgery, colorectal cancer, complications, length of hospital stay

## Abstract

Enhanced Recovery After Surgery (ERAS) protocols have changed perioperative care, aiming to optimize patient outcomes. This study assesses ERAS implementation effects on postoperative complications, length of hospital stay (LOS), and mortality in colorectal cancer (CRC) patients. A retrospective real-world analysis was conducted on CRC patients undergoing surgery within a Northern Italian Cancer Registry. Outcomes including complications, re-surgeries, 30-day readmission, mortality, and LOS were assessed in 2023, the year of ERAS protocol adoption, and compared with data from 2022. A total of 158 surgeries were performed, 77 cases in 2022 and 81 in 2023. In 2023, a lower incidence of postoperative complications was observed compared to that in 2022 (17.3% vs. 22.1%), despite treating a higher proportion of patients with unfavorable prognoses. However, rates of reoperations and readmissions within 30 days post-surgery increased in 2023. Mortality within 30 days remained consistent between the two groups. Patients diagnosed in 2023 experienced a statistically significant reduction in LOS compared to those in 2022 (mean: 5 vs. 8.1 days). ERAS protocols in CRC surgery yield reduced postoperative complications and shorter hospital stays, even in complex cases. Our study emphasizes ERAS’ role in enhancing surgical outcomes and recovery.

## 1. Introduction

The concept of Enhanced Recovery After Surgery (ERAS) represents a paradigm shift in perioperative care aiming to optimize patient outcomes through a comprehensive, multidisciplinary approach. ERAS protocols encompass a spectrum of interventions spanning the preoperative, intraoperative, and postoperative phases, all strategically designed to mitigate the physiological stressors associated with surgery while promoting expedited recovery [1,2,3]. This approach has gained traction across various surgical disciplines, including urology, gynecology, and gastrointestinal surgery [3,4,5,6]. However, the most robust evidence supporting the efficacy of ERAS protocols stems from studies within the realm of colorectal surgery [7,8]. Research has consistently demonstrated the significant benefits conferred by ERAS pathways in patients undergoing elective colorectal surgery, manifesting as a reduction in postoperative complications and length of hospital stay (LOS) [9,10,11]. Furthermore, the integration of laparoscopy, as the gold standard technique, with ERAS protocols has emerged as a cornerstone in the management of colorectal diseases [12].

Despite being described over a quarter of a century ago by Kehlet et al. [13,14], and notwithstanding the evident advantages conferred by ERAS, its widespread adoption has been hampered by pragmatic hurdles, chiefly the necessity for surgeons to diverge from established perioperative conventions that have historically yielded predictable outcomes [15]. Consequently, the median hospital stay following major colorectal surgery remains substantial, with an average duration of 6 days, ranging from 7 to 10 days [16,17]. Given the ongoing evolution and refinement of ERAS protocols, there persists a need for robust evaluation of their impact on patient outcomes. Accordingly, the objective of the present study was to describe the effects of the ERAS procedure on patients diagnosed with CRC between 2022 and 2023 within a province in northern Italy.

Specifically, this investigation aims to assess outcomes such as postoperative complications, 30-day mortality rates, and LOS, thereby contributing to the growing body of literature informing perioperative care practices.

## 2. Materials and Methods

### 2.1. Study Setting

The Reggio Emilia (RE) Cancer Registry (CR) serves a population of 532,000 inhabitants and has been operational since 1996, consistently collecting up-to-date data, with incidence records extending to the end of 2021. It boasts a notably high rate of microscopic confirmation (93.2% for CRC) and minimal reliance on Death Certificate (DCO) registration, accounting for less than <0.1% of patients [18]. The CR diligently collects and analyzes data according to prevailing protocols to generate statistics on cancer incidence, mortality, prevalence, and survival rates for the resident population and demographic subgroups, as required by the epidemiological report outlined in Law no. 29 of 03/22/2019, which governs the CR in Italy. Notably, this legislation exempts CR from the obligation to obtain informed consent for data collection purposes. The procedures governing the epidemiological analyses of data collected by the RE–CR have received approval from the provincial Ethics Committee of Reggio Emilia (Protocol no. 2014/0019740 of 04/08/2014).

### 2.2. Data Sources

The leading data sources utilized by the RE–CR comprise pathological reports, hospital discharge records, and mortality data, which are further integrated with laboratory tests, diagnostic reports, and inputs from general practitioners. The data presented here were not drawn from representative samples, nor selected from large case studies. In this study, we only reported the data relating to patients with colorectal cancer registered in the province of Reggio Emilia in the two periods considered. Therefore, the variables were not selected a priori, but were only described as they occur in the real world.

Following the implementation of the ERAS protocol in March 2023, the study encompassed all patients undergoing CRC surgery between March and October 2023, compared with the corresponding data from the equivalent timeframe in 2022. Case identification was predicated on the International Classification of Diseases for Oncology, Third Edition (ICD-O-3) with a focus on topography classified under C18-C19 [19]. Comprehensive details on disease staging (TNM 8th edition) [20], surgical procedures, therapeutic modalities, and postoperative complications were extracted from hospital medical records.

### 2.3. ERAS Protocol

The operational protocol for the ERAS procedure is delineated in Table 1. Comprising three distinct phases—preoperative, intraoperative, and postoperative—the ERAS protocol is structured to optimize patient outcomes through the attenuation of surgical stress [13,14] across these key perioperative periods. The initial phase, commonly termed prehabilitation, pertains to the period preceding surgery, during which both the surgeon and the anesthetist intervene to address factors contributing to recovery, encompassing physical and psychological aspects, thereby alleviating emotional distress associated with the surgical anticipation and recovery [21,22,23,24]. Preoperative counseling plays a pivotal role in conveying the nuances of the ERAS protocol. Surgeons typically advise patients to cease smoking and alcohol consumption while encouraging engagement in physical activity. Thromboembolism prophylaxis and bowel preparation, along with oral antibiotic prophylaxis, are administered preoperatively. Patients are encouraged to initiate the intake of specific nutrients five to seven days before surgery, tailored to their nutritional status. Additionally, all patients are instructed to consume maltodextrin the evening prior to surgery and the two hours preceding incision.

The ERAS protocol can be implemented in various surgical approaches, including both open surgery and minimally invasive laparoscopic surgery. Following the procedure, the nasogastric tube is typically removed in the operating room. Patients are encouraged to start mobilization within 4–6 h post-surgery, thus promoting early ambulation. Initiation of oral fluid intake typically starts on the same day as surgery, facilitating hydration and promoting gastrointestinal function. Furthermore, a soft diet is usually introduced from the first day after surgery (POD1), aiding in nutritional support and gastrointestinal comfort. Urinary catheter removal typically occurs on the morning of POD1 (removal of the bladder catheter is expected within 24–48 h even in rectal surgery, subject to confirmation of valid diuresis), promoting the early restoration of bladder function and reducing the risk of urinary tract complications.

Finally, pain management strategies aim to minimize opioid usage, where feasible, with a preference for non-opioid analgesics, such as paracetamol or nonsteroidal anti-inflammatory drugs (NSAIDs).

All perioperative data were recorded in a comprehensive database, facilitating systematic analysis and evaluation. Postoperative complications were classified using the Clavien–Dindo classification [25], which provides a standardized framework for characterizing the severity of postoperative complications. Additionally, the American Society of Anesthesiologists (ASA) Physical Status Classification System was utilized to stratify patients based on preoperative comorbidities and overall health status, enabling risk assessment and tailored perioperative management strategies [26].

### 2.4. Statistical Methods

Descriptive statistics were computed for various demographic and clinical variables, including gender, ASA classification, tumor site (ascending colon, transverse colon, descending colon, rectum), cancer stage, surgical approach (laparoscopy or laparotomy), administration of chemotherapy and radiotherapy, body mass index (BMI), and age at diagnosis, stratified by year of surgery (2022 vs. 2023). Furthermore, outcomes, such as postoperative complications leading to re-surgery within 30 days, readmission within 30 days, mortality within 30 days, duration of hospitalization, and the Clavien–Dindo classification, were assessed. Fisher’s exact test, the χ2-test, and the t-test, as appropriate, were performed to evaluate differences between the two years of surgery. We also calculated the odds ratio (OR) with a relative 95% confidence interval (CI) using logistic regression analysis to assess the impact of complications on the ERAS procedure and other possible predictors such as sex, age at diagnosis, BMI, and stage. Analyses were performed using STATA 16.1 software. A significance level of a *p*-value < 0.05 was considered statistically significant.

## 3. Results

**Figure 1:**
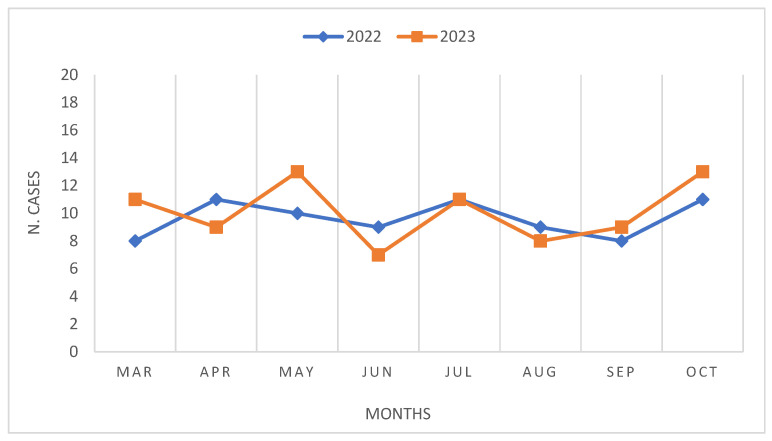
Reggio Emilia Cancer Registry 2022–2023. Comparison of number of surgeries by month.

During the period spanning March to October of both 2022 and 2023, a total of 158 surgeries for CRC were conducted, with one case of ovarian cancer with intestinal involvement. Specifically, 77 surgeries were performed in 2022, while 81 surgeries were conducted in 2023 (Table 2, Figure 1). Notably, in 2023, there was a lower proportion of male patients undergoing surgery compared to that in 2022 (51.8% vs. 59.7%), along with a higher incidence of patients classified as ASA 3 (44.4% vs. 35.1%), and those with tumors located in the descending colon (27.2% vs. 16.9%). Regarding the stage of cancer at diagnosis, 2023 witnessed a higher proportion of patients undergoing surgery at stage I (25.9% vs. 20.8%), albeit accompanied by an increase in the number of patients diagnosed at stage IV (17.3% vs. 11.7%). Furthermore, laparoscopic procedures were significantly more prevalent in 2023, accounting for 92.6% of surgeries, compared to 80.5% in 2022 (*p*-value < 0.05). In 2023, there was a higher frequency of patients receiving neoadjuvant chemotherapy but a lower incidence of patients receiving radiation therapy. No significant differences were observed between the two groups in terms of BMI with mean values of 26.5 in 2023 and 25.4 in 2022. However, there was a notable disparity in the mean age at diagnosis between the two cohorts, with patients in 2023 being older on average (72.2 vs. 68.1 years; *p*-value < 0.05).

The majority of the surgeries were conducted with a single resection (right colectomy, left colectomy, an anterior resection of the rectum -RAR-, an abdominal–perineal resection of the rectum –Miles-, resection of the transversus, a resection of the splenic flexure, and Hartmann’s procedure), but a total of 24 multivisceral resections were performed during both periods. Twelve multivisceral resections were performed in 2023 and twelve multivisceral resection in 2022. The most common multivisceral resections were a colic resection with a hepatic resection, a kidney resection, or an ovariectomy. In some cases, a bladder resection, adrenalectomy, or hysterectomy was performed (Tables S1 and S2).

In 2023, a lower incidence of postoperative complications was recorded compared to that in 2022 (17.3% vs. 22.1%), whereas there were higher rates of reoperations (6.2% vs. 3.9%) and readmissions (8.6% vs. 3.9%) recorded within 30 days following surgery (Table 3). Notably, there were no discernible differences in terms of 30-day mortality between the two cohorts, with one death recorded in each group. However, patients diagnosed in 2023 exhibited a statistically significant reduction in days of hospitalization (5 days) compared to those in 2022 (8.1 days) (*p*-value < 0.01).

The rate of complications recorded using the Clavien–Dindo scale (Table 4) indicates that in 2023, 50% of minor complications (CD I and II) and 50% of major complications were reported, which represents an increase compared to the rate observed in 2022 (24%), even if not statistically significative. Specifically, the seven patients who experienced CD III-IV complications in 2023 included cases of bleeding (two of which were managed with endoscopic hemostasis and one with surgical hemostasis), intrabdominal abscess, colic ischemia in the setting of Clostridium difficile infection, and medical complications (vascular and neurological). In contrast, the four patients in 2022 experienced complications such as bowel obstruction, hernia, and liver failure (Table S3).

A multivariate analysis (Table 5) confirmed that in 2023 a decrease in mortality was observed, although not significant (OR 0.64; 95% CI 0.26–1.59) in general but only in females (OR 0.34; 95% CI 0.13–0.89). An excess risk, however, was confirmed for a BMI > 30 (OR 5.62; 95% CI 1.68–18.81) and, although not significative, for more advanced stages.

## 4. Discussion

The application of ERAS procedures promotes the early restoration of patients’ homeostasis following colorectal surgery [27]. Patients begin early mobilization and walking, receive early fluid and oral diets, and have intravenous infusions interrupted promptly [8,15]. This comprehensive approach leads to a faster restoration of physiological homeostasis and facilitates the overall recovery process for the patient, playing a pivotal role in facilitating faster recovery and reducing postoperative complications.

The findings of our study provide valuable insights into the impact of implementing the ERAS protocol on patients undergoing CRC surgery in the period spanning March to October of 2022 and 2023.

First and foremost, one of the salient findings from our analysis was the notable increase in laparoscopic procedures observed in 2023. These procedures accounted for 92.6% of surgeries during this period, representing a substantial rise compared to the 80.5% recorded in 2022. This shift towards laparoscopic approaches underscored the growing acceptance and adoption of minimally invasive techniques in colorectal surgery, reflecting advancements in surgical technology and techniques. The ERAS approach introduced in 2023 appeared to be able to reduce hospital days and, although not significantly, the appearance of complications. Additionally, the higher proportion of patients undergoing surgery for stage I disease in 2023 suggests potential improvements in early detection and diagnostic practices, possibly facilitated by enhanced screening efforts or advancements in imaging technology.

Secondly, our study revealed that despite treating a greater number of patients with an unfavorable prognosis in 2023, including more ASA-3-case classifications, advanced stages, and an older age, the activation of the ERAS procedure led to fewer complications and shorter hospital stays, suggesting that the ERAS protocol could effectively mitigate the adverse effects of surgery and promote faster recovery, even in patients with more complex medical profiles.

Our study findings were consistent with existing literature, demonstrating the favorable outcomes associated with ERAS protocols in colorectal surgery patients. The average LOS in our study was 5 days, which aligned with other studies reporting similar reductions in hospitalization duration following the implementation of ERAS protocols [10,28]. Moreover, the concept of ‘fast-track’ surgery, often synonymous with ERAS, has been shown to significantly shorten hospital stays in colorectal surgery patients. Meta-analyses of several randomized controlled trials (RCTs) comparing traditional perioperative care with fast-track surgeries have consistently reported shorter hospital stays [3,29,30,31,32]. Additionally, single-institution observational studies have described reductions in LOS ranging from 1 to 3 days following the introduction of ERAS protocols [33,34,35].

Analyses of the reoperation cases in both years highlighted the diverse nature of complications encountered, ranging from hernias and bowel obstructions to bleeding and colic ischemia, showcasing the complexity and multifactorial nature of postoperative complications in colorectal surgery patients. Similarly, readmissions were driven by various factors such as abdominal abscesses and Clostridium difficile infections. Noteworthily, our study unveiled an increase in reoperation and readmission rates at 30 days post-surgery in 2023, despite the application of ERAS protocols. Further investigation is warranted to elucidate the underlying reasons for this increase.

Determining which complications are worse can be challenging and often depends on various factors such as the severity of the complication, its impact on patient morbidity and mortality, and the required interventions for management. In 2023, the patients with major complications (Clavien–Dindo III-V) were as follows: Two patients had some bleeding from the anastomosis that was successfully stopped through an endoscopic exam with applications of hemostatic clips. One patient was complicated with an abscess on the site of surgery, but the abscess was present also before surgery because of a perforation of the cancer in the abdomen. We performed a radiologic drainage to clean the abscess and to avoid more dangerous septic complications for the patient. Another patient went to the operating room a few hours after the first surgery because of bleeding; during the first surgery, we performed a right colic resection with a right nephrectomy and duodenal transversal resection. One patient was re-operated on for colic ischemia after a left colectomy with a hepatic resection during the first surgery. All the patients with major complications were treated conservatively, except two of them who underwent surgery.

The patients affected by CD V died from medical complications: the first one had an ASA score of 4, and after the surgery (right colectomy) was affected by neurological and vascular complications that led to mortality. The second one had an ASA score of 3, and after two re-interventions for bowel subocclusion the first time and for bowel perforation the second time, he was transferred to the Intensive Care Unit and then the recovery was complicated by a significant thalamic hemorrhage that led to mortality. All the patients complicated in the 2023 series were ASA IV or III.

Overall, two deaths occurred within 30 days of surgery, one in 2022 and the other in 2023. In 2022, one patient with cirrhosis from liver failure, while in 2023, one patient from neurological and vascular causes.

These findings suggest that ERAS protocols appear capable of mitigating some complications and accelerating post-surgery recovery, even if they fail to fully address factors contributing to reoperations and readmissions. Another critical aspect to highlight concerns intestinal manipulation. Recent research has shown variable rates of reinfections following colorectal surgery, ranging from 5.4% to 23.2%, with a weighted average of 11.4% [36]. The American Society of Colon and Rectal Surgeons clinical practice guidelines recommend mechanical bowel preparation (MBP) combined with preoperative oral antibiotics for elective colorectal resections [37]. Despite conflicting evidence regarding the need for bowel preparation, our institution chose to reintroduce MBP before surgery because a clean colon is believed to reduce the risk of contamination during that process.

This approach is in line with emerging evidence suggesting a potential reduction in surgical site infections when MBP is combined with preoperative oral antibiotics and meticulous perioperative antibiotic prophylaxis [38,39,40].

Indeed, another concern worth highlighting is the care of elderly patients undergoing colorectal surgery. As the population continues to age, the number of elderly patients requiring surgical intervention for colorectal conditions is expected to rise. Elderly patients present unique challenges due to age-related physiological changes, increased susceptibility to postoperative complications, and higher rates of comorbid conditions. Initially, ERAS protocols were perceived as suitable only for young and healthy patients. However, our study challenged this notion. Our experience seemed to confirm a slight decrease in complications and hospital days in 2023, even if these were patients who were ill-fitting and had comorbidities [41,42,43].

By standardizing perioperative care pathways and optimizing resource utilization, the adoption of ERAS protocols represents a prudent investment that can yield economic benefits and enhance the sustainability of healthcare delivery [44].

Finally, our study drew strength from its utilization of population-based data and recent observations. However, its reliance on a single-center design imposed limitations. Future investigations should prioritize replication in multicenter studies to enhance the validation of ERAS protocols’ efficacy and applicability in colorectal cancer surgery. Additionally, the current absence of qualitative data on patients’ quality of life underscores a gap in our understanding that warrants further exploration.

## 5. Conclusions

The ERAS program, pioneered by Kehlet in 1999 [13,14] to mitigate surgical stress and expedite postoperative recovery, has emerged as the gold standard in colorectal surgery. Our study, even if referring to a small population of patients, seemed to confirm in 2023 a decrease in hospital days and, although not significant, the probability of having complications, especially in women, while worse outcomes were associated with age, stage, and an increase in BMI.

## Figures and Tables

**Table 1:** ERAS protocol modification at the Surgical Oncology Unit, Reggio Emilia, Azienda, USL-IRCCS.

**Preoperative phase**	
Preoperative counseling	- Patients were evaluated by the Multidisciplinary Oncological Group and adequately informed to ameliorate adherence to the protocol.
Bowel preparation	- Oral Macrogol consumption.
Thromboembolism prophylaxis	- Low molecular weight heparins.
	- Paromomycin and Metronidazole per os during the 48 h before the surgery in association with the bowel preparation.
Antibiotic prophylaxis	- Penicillin or cephalosporins was used at induction.
**Intraoperative phase**	
Minimally invasive surgery (in a few cases, open surgery)	- Laparoscopic surgery with intracorporeal or extracorporeal anastomosis with small incision.
	- No drainage use.
**Postoperative phase**	
Nasogastric tube	- Removed after surgery in the operating room.
Pain control	- Paracetamol or NSAI drugs i.v., opioids as 3rd choice.
Early mobilization	- It begins 4 h after the intervention. Walking encouraged from POD (post operative day) 1.
Oral fluid and food intake	- Oral fluid intake begins the same day and a soft diet from POD1.
Urinary catheter removal	- Upon awaking or POD1.

**Table 2:** Reggio Emilia Cancer Registry 2022–2023. Characteristics of patients by sex, ASA, site, stage, treatments, BMI, and age at diagnosis.

	All		2022		2023		*p*-Value
	n	%	n	%	n	%	
Overall	158		77		81		
Sex							
Female	70	44.3	31	40.3	39	48.2	0.3
Male	88	55.7	46	59.7	42	51.8	
ASA							
1	11	7.0	7	9.1	4	4.9	0.3
2	76	48.1	37	48.0	39	48.2	
3	63	39.9	27	35.1	36	44.4	
4	8	5.0	6	7.8	2	2.5	
Site							
Ascending colon	70	44.3	36	46.8	34	42.0	0.5
Transverse colon	9	5.7	5	6.5	4	4.9	
Descending colon	35	22.2	13	16.9	22	27.2	
Rectum	44	27.8	23	29.9	21	25.9	
Stage							
I	37	23.4	16	20.8	21	25.9	0.4
II	42	26.6	25	32.5	17	21.0	
III	56	35.4	27	35.1	29	35.8	
IV	23	14.6	9	11.7	14	17.3	
Surgery							
Laparoscopy	137	86.7	62	80.5	75	92.6	<0.05
Laparotomy	21	13.3	15	19.5	6	7.4	
Neo Chemotherapy							
Yes	31	19.6	11	14.3	20	24.7	0.1
No	127	80.4	66	85.7	61	75.3	
Neo Radiotherapy							
Yes	27	17.1	14	18.2	13	16.1	0.7
No	131	82.9	63	81.8	68	83.9	
	**mean**	**sd**	**mean**	**sd**	**mean**	**sd**	
BMI	26.0	5.0	25.4	4.7	26.5	5.3	0.08
Age at diagnosis	70.0	14.2	68.1	15.6	72.2	12.4	<0.05

**Table 3:** Reggio Emilia Cancer Registry 2022–2023. Patient outcomes in terms of complications, reoperations, death, and hospital stay.

	2022		2023		
	n	%	n	%	*p*-Value
Overall	77		81		
Complications					
Yes	17	22.1	14	17.3	0.5
No	60	77.9	67	82.7	
Re-surgery in 30 days					
Yes	3	3.9	5	6.2	0.5
No	74	96.1	76	93.8	
New entry in 30 days					
Yes	3	3.9	7	8.6	0.2
No	74	96.1	74	91.4	
Death in 30 days					
Yes	1	1.3	1	1.2	0.9
No	76	98.7	80	98.8	
	**mean**	**sd**	**mean**	**sd**	
Days of hospitalization	8.1	5.0	5.0	3.5	<0.01

**Table 4:** Reggio Emilia Cancer Registry 2022–2023. Type of complications, using the Clavien–Dindo classification.

Clavien–Dindo	2022		2023		Total		
	n	%	n	%	n	%	*p*-Value
I	6	35.3	3	21.4	9	29.0	0.40
II	7	41.2	4	28.6	11	35.5	0.47
III	3	17.7	5	35.7	8	25.8	0.25
V	1	5.9	2	14.3	3	9.7	0.43
Total	17		14		31		

**Table 5:** Logistic regression adjusted for ERAS status, age, BMI, gender, stage, and surgery.

	Univariable Analysis			Multivariable Analysis		
	OR	95% CI		OR	95% CI	
Year of surgery						
2022	1.0	Ref.		1.0	Ref.	
2023	0.74	0.34	1.62	0.64	0.26	1.59
Age	1.00	0.98	1.03	1.01	0.98	1.05
BMI						
≤18.5	0.64	0.07	5.82	0.67	0.06	6.92
18.6–24.9	1.0	Ref.		1.0	Ref.	
25.0–29.9	1.07	0.39	2.95	0.99	0.35	2.82
≥30.0	4.49	1.46	13.84	5.62	1.68	18.81
Gender						
Male	1.0	Ref.		1.0	Ref.	
Female	0.36	0.16	0.88	0.34	0.13	0.89
Stage						
I	1.0	Ref.		1.0	Ref.	
II	1.34	0.45	3.97	1.11	0.33	3.73
III	0.82	0.28	2.43	1.07	0.33	3.49
IV	1.19	0.33	4.31	1.42	0.34	5.88
Surgery						
Laparotomy	1.0	Ref.		1.0	Ref.	
Laparoscopic	0.75	0.25	2.23	0.65	0.20	2.12

## Data Availability

The data presented in this study are available on request from the corresponding author. The data are not publicly available due to ethical and privacy issues, requests of data should be approved by the Ethic Committee after the presentation of a study protocol.

## Supplementary Materials

The following supporting information can be downloaded at: https://www.mdpi.com/article/10.3390/curroncol31060222/s1, Table S1: Reggio Emilia Cancer Registry 2022–2023. Type of surgery, comparison per year; Table S2: Reggio Emilia Cancer Registry 2022–2023. Type of multivisceral resections, comparison per year.; Table S3: Reggio Emilia Cancer Registry 2022–2023. Type of major complication after surgery, per year.
