# Supplementary material for: Outcomes before and after Implementation of the ERAS (Enhanced Recovery after Surgery) Protocol in Open and Laparoscopic Colorectal Surgery: A Comparative Real-World Study from Northern Italy
Source: Curr Oncol. 2024 May 21;31(6):2907–17. doi: 10.3390/curroncol31060222 (PMC11202664; doi:10.3390/curroncol31060222)
Supplement: Supplementary file 1 [file curroncol-31-00222-s001.zip › curroncol-2991648-supplementary.pdf]

Supplementary Tables

Table S1. Reggio Emilia Cancer Registry 2022-2023. Type of surgery, comparison per year.

Type of surgery	2022	2023	Total
Total colectomy	0	1	1
Right colectomy	30	28	58
Left colectomy	7	14	21
Anterior Rectal resection	13	20	33
Miles	5	3	8
Resection of trasversus	4	2	6
Resection of splenic flexure	2	1	3
Hartmann	4	0	4
Multivisceral resection	12	12	24
	**77**	**81**	**158**

Table S2. Reggio Emilia Cancer Registry 2022-2023. Type of Multivisceral resections, comparison per year.

Type of surgery			
**Cases**	**2022**	**Cases**	**2023**
1	Right colectomy + sigmoid resection	1	Right colectomy+ovariectomies
2	Right colectomy + left colectomy	2	Right colectomy +hepatic resection
3	Hartmann procedure + bladder resection	3	Right colectomy +hepatic resection
4	Right colectomy+ right adrenalectomy	4	RAR +right colectomy
5	Miles +isterectomy	5	Left colectomy +hepatic resection
6	Left colectomy+ hepatic resection	6	Pelvic exenteration
7	Right colectomy+ ovariectomy	7	Right colectomy+ left colectomy
8	Resection of colon trasversus+ gastric wedge resection	8	Left colectomy + transurethral removal of papilloma of the bladder
9	Miles+ cystectomy	9	RAR+ partial cystectomy
10	Right colectomy+ hepatic resection	10	Left colectomy + partial resection of il-eum
11	Right colectomy+ hepatic resection	11	Right colectomy + right nephrectomy + transversal resection of duodenum
12	RAR+ hysterectomy	12	Proctectomy +total gastrectomy

Table S3. Reggio Emilia Cancer Registry 2022-2023. Type of Major complication after surgery, per year.

Clavien-Dindo	Complication	Treatment
**2022**		
III	bowel obstruction	surgery
III	bowel obstruction	surgery
III	hernia in the site of a previous trocar	surgery
V	liver failure	medical treatment
**2023**		
III	bleeding from the anastomosis	endoscopic coagulation
III	bleeding from the anastomosis	endoscopic coagulation
III	abscess at the site of surgery	radiologic drainage
III	bleeding	surgical hemostasis
III	colic ischemia in C. Difficile’s infection	Surgical colic resection
V	Neurological and vascular complication	ICU treatment
V	Colic perforation, thalamic hemorrhage	ICU treatment
